# Higher Blood Urea Nitrogen and Urinary Calcium: New Risk Factors for Diabetes Mellitus in Primary Aldosteronism Patients

**DOI:** 10.3389/fendo.2020.00023

**Published:** 2020-02-04

**Authors:** Yu Liu, Liang Zhou, Zhenghuan Liu, Yucheng Ma, Lede Lin, Yuchun Zhu, Kunjie Wang, Hong Li

**Affiliations:** Department of Urology, Institute of Urology, West China Hospital, Sichuan University, Chengdu, China

**Keywords:** primary aldosteronism, diabetes mellitus, risk factors, blood urea nitrogen, urinary calcium

## Abstract

**Purpose:** The aim of the study was to investigate the prevalence and risk factors of diabetes mellitus (DM) in primary aldosteronism (PA) patients.

**Methods:** This case-control study enrolled 259 PA patients in West China Hospital, China from January 2016 to January 2019. Patients were divided into three groups: PA group, PA + impaired fasting glucose (IFG)/impaired glucose tolerance (IGT) group and PA + DM group. Clinical characteristics (like age and sex) and laboratory variables (like plasma aldosterone concentration and plasma renin activity) were compared between three groups. Univariate and multivariate logistic regression analyses were performed to determine risk factors for DM in PA patients. The association of random blood glucose with the above-mentioned factors were also investigated by Pearson correlation analyses. Nomogram model was developed to predict the probability of DM in PA patients.

**Results:** 49 (18.9%) patients were diagnosed with DM and 22 (8.5%) with IFG/IGT in 259 PA patients. Apart from older age, male, higher body mass index, higher triglycerides and lower cholesterol, we found that higher blood urea nitrogen (BUN) and higher 24 h urinary calcium (Ca) might be potential new risk factors for dysglycemia. The nomogram model for DM in PA patients had a good predictive accuracy, with the area under the curve of receiver operating characteristic of 0.839 (95% CI 0.784–0.893).

**Conclusions:** PA patients were more likely to have DM compared with general population. Apart from older age, overweight and dyslipidemia, higher BUN and excessive excretion of urinary Ca may also be the new potential risk factors for DM in PA patients.

## Introduction

Primary aldosteronism (PA) is a disease accompanied with hypertension and hypokalemia caused by excessive secretion of aldosterone from the adrenal glands. Recent epidemiological studies found that it was not rare in hypertension patients, with prevalence ranging from 5 to 10% (1, 2). The recommended treatment is adrenalectomy for unilateral PA (3). Wu et al. reported that the incidence of new-onset diabetes mellitus (DM) in PA patients who received adrenalectomy (12.7/1,000) was much lower than that in essential hypertension (EH) controls (28.1/1,000) (4). PA patients also showed an increase of insulin secretion and insulin sensitivity after adrenalectomy (5–7). In addition, it was also shown that the mean blood glucose, insulin sensitivity and percentage of DM were significantly higher in PA patients than those with EH (8, 9).

All the evidence above mentioned suggested that PA may be associated with impaired glucose homeostasis. Some scientists regarded high serum aldosterone as a risk factor for DM (10–12). However, others did not find any association between PAC and DM (9, 13, 14). Additionally, Watanabe et al. reported a negative correlation between serum potassium and insulin sensitivity (15). And it was confirmed by some studies which showed lower serum potassium concentration in PA patients with DM (11, 12). However, some studies also showed that low serum potassium may not contribute to the development of DM (9, 10, 13). At present, the underlying contributors to DM in PA patients were still not quite clear. Thus, we wanted to investigate the prevalence and risk factors of DM in PA patients. In addition, we also wanted to build a predictive model for DM in PA patients based on the risk factors using nomogram. Using this model, physicians may tell which kind of PA patients were more likely to develop DM and take actions in advance to reduce the possibility of DM in the future.

## Materials and Methods

### Study Design

This case-control study was conducted to identify the risk factors for DM in PA patients. It was performed according to the strengthening the reporting of observational studies in epidemiology guideline.

### Study Population

This retrospective study enrolled all the PA patients who had received adrenalectomy in Urology Department, West China Hospital, China from January 2016 to January 2019. The minimal sample size was calculated using online sample size calculator (http://powerandsamplesize.com/Calculators/). We chose “Test 1 Proportion: 1-Sample, 2-Sided Equality” and the parameters were set as follows: power (1-β) = 0.80, error rate (α) = 0.05, true proportion (*p*) = 0.17, and null hypothesis proportion (p0) = 0.07. The calculated minimal sample size was 148. Patients were divided into three groups: PA group, PA + impaired fasting glucose (IFG)/impaired glucose tolerance (IGT) group and PA + DM group. PA was diagnosed by the following three steps according to the American Endocrine Society guideline 2016 for PA (3). Firstly, the plasma aldosterone-to-renin ratio (ARR) more than 30 might indicate possible PA. Then, PA was confirmed by two confirmatory tests, including captopril test and saline infusion test. Finally, adrenal computed tomography and adrenal venous sampling were performed to distinguish between unilateral and bilateral adrenal tumor. The diagnosis of DM, IFG, and IGT were referred to the latest clinical guideline for DM in China (16). DM was confirmed if any of the following conditions was met: random blood glucose (RBG)/120-minute oral glucose tolerance test (OGTT120) ≥ 11.1 mmol/L, fasting plasma glucose (FPG)/0-minute oral glucose tolerance test (OGTT0) ≥7.0 mmol/L, glycosylated hemoglobin ≥6.5%. IFG was defined as 6.2 mmol/L ≤ FPG/OGTT0 ≤ 6.9 mmol/L and RBG/OGTT120 ≤ 7.7 mmol/L. IGT was regarded as 7.8 mmol/L ≤ RBG/OGTT120 ≤ 11.0 mmol/L and FPG/OGTT0 ≤ 6.1 mmol/L.

### Data Collection

The data for the study was obtained from the database of our hospital. We retrospectively reviewed patients' medical records from the time when they were diagnosed as PA to their last visit to our department. All the following possible risk factors were extracted.

Clinical characteristics included age, sex, body mass index (BMI), duration of hypertension, the maximal systolic blood pressure (SBP), side of tumor, the maximal diameter of tumor, dizziness or headache, palpitation, weakness or acroanesthesia. BMI was calculated by dividing weight in kilogram by the square of height in meter.

The following laboratory variables were also collected. Glucose metabolic factors included RBG, OGTT0, OGTT120, and glycosylated hemoglobin. Lipid metabolic factors were triglycerides (TG), total cholesterol (CHOL), high density lipoprotein (HDL), and low density lipoprotein (LDL). Parameters of renin-angiotensin-aldosterone system of different position (lying or standing position) were PAC, plasma renin activity, and ARR. Parameters associated with metabolism of glucocorticoid were as follows: plasma total cortisol at 0 am and 8 am, adrenocorticotropin, total 24 h urine free cortisol and its concentration. Renal function indices were serum creatinine, estimated glomerular filtration rate (eGFR), uric acid, and blood urea nitrogen (BUN). Additionally, the concentration of serum and urinary electrolytes as well as total 24 h urinary electrolyte were also extracted, including potassium, sodium, chlorine, calcium (Ca), magnesium (Mg), and phosphorus (P).

Most biochemical factors were examined using Roche Cobas 8000. Specifically, serum and urinary electrolytes were analyzed using ion selective electrode, glucocorticoid and cortisol using electrochemiluminescence, lipid metabolic factors using enzymatic colorimetric assay, and blood glucose using hexokinase method. Renin, angiotensin and aldosterone were measured using radioimmunoassay.

### Statistical Analysis

Samples were only excluded if they lack the value of some factors when comparing specific factor between groups. Continuous variables which obeyed normal distribution were shown as mean and standard deviation. Otherwise, median and inter-quartile range were considered. Differences in continuous variables were evaluated using 2-sided analysis of variance or Kruskal-Wallis rank sum test based on their distribution. Categorical variables were presented as counts and percentages, and differences in them were evaluated using Chi-square test. Next, we screened out potential risk factors for DM using univariate and multivariate logistic regression analyses. Odds ratios and 95% confidence intervals (CI) were calculated. Multivariate logistic regression analyses were adjusted for age, sex, BMI, TG, CHOL, HDL, and LDL, which were already defined as risk factors for DM in general population. Similarly, PA patients were divided into two groups: PA group and PA + DM/IGT/IFG group. And logistic regression analyses were again performed between these two groups. Pearson correlation analysis was also performed to explore the relationship between some factors. We also divided PA patients into two parts based on the level of the potential risk factors above mentioned and compared the percentage of DM.

Finally, based on these potential risk factors, we developed a nomogram model to predict the probability of having DM in PA patients. The predictive accuracy of the nomogram was assessed by area under the curve of receiver operating characteristic (ROC AUC) and 95% CI. We used all the 259 samples as training cohort and test cohort. Then, internal 10-fold cross validation was also performed. Briefly, 259 samples were divided into 10 parts randomly. Each part was used as test cohort and the remaining parts as training cohorts. The mean ROC AUC was calculated based on the ten values. Calibration curve was used to assess how well the actual percentage of DM matched the predicted one. In addition, we also used decision curve analysis to evaluate the net benefits when actions were taken to prevent the development of DM.

*P* < 0.05 was defined as statistical significance. All the data were collected using Microsoft Excel and analyzed using IBM SPSS statistics software. Pearson correlation analysis was visualized with R 3.6.0 and package ggplot2. Nomogram was developed using R packages, including rms, nomogram Ex, caret, and MASS.

## Results

### Characteristics of PA Patients With or Without DM/IFG/IGT

Clinical characteristics and laboratory test results were shown in Table 1. A total of 259 PA patients who had underwent adrenalectomy were enrolled in the study. Before surgery, 49 (18.9%) patients were also diagnosed with DM and 22 (8.5%) with IFG/IGT. Among DM patients, 73.5% (36/49) of them were diagnosed with hypertension before DM. The mean age ± standard deviation of all the PA patients was 47.00 ± 12.28 years and 44.0% were male. The mean BMI ± standard deviation was 24.28 ± 3.53 kg/m^2^. Most tumors (61.1%) were on the left adrenal gland. The most common symptom was weakness or acroanesthesia (56.8%), followed by dizziness or headache (52.2%) and palpitation (22.8%).

**Table 1:** Characteristics of the PA patients with or without DM/IFG/IGT.

	**Factors**	**Total (*n* = 259)**	**PA (*n* = 188)**	**PA+IFG/IGT (*n* = 22)**	**PA+DM (*n* = 49)**	***P*-value**
Clinical characteristics	Age (years)	47.00 ± 12.28	44.63 ± 11.97	50.68 ± 11.06	54.41 ± 10.68	**<0.001**^(a)^
	Sex (male/female)	114 (44.0)/145 (56.0)	72 (38.3)/116 (61.7)	11 (50.0)/11 (50.0)	31 (63.3)/18 (36.7)	**0.006**^(c)^
	BMI (kg/m^2^)	24.28 ± 3.53	23.52 ± 3.21	24.61 ± 4.14	26.99 ± 3.05	**<0.001**^(a)^
	Duration of hypertension (months)	60.00 (108.00)	48.00 (96.00)	60.00 (99.00)	96.00 (126.00)	**0.002**^(b)^
	Maximal SBP (mm Hg)	180.00 (31.00)	180.00 (33.00)	181.00 (30.00)	180.00 (33.00)	0.301^(b)^
	Location of tumor (left/right/bilateral)	157 (61.1)/92 (35.8)/8 (3.1)	116 (62.0)/67 (35.8)/4 (2.1)	15 (68.2)/7 (31.8)/0 (0)	26 (54.2)/18 (37.5)/4 (8.3)	0.186^(c)^
	Maximal diameter of tumor (cm)	1.50 (0.70)	1.60 (0.80)	1.60 (0.68)	1.30 (0.73)	0.074^(b)^
	Dizziness/headache (no/yes)	123 (47.5)/136 (52.2)	88 (46.8)/100 (53.2)	11 (50.0)/11 (50.0)	24 (49.0)/25 (51.0)	0.935^(c)^
	Palpitation (no/yes)	200 (77.2)/59 (22.8)	148 (78.7)/40 (21.3)	15 (68.2)/7 (31.8)	37 (75.5)/12 (24.5)	0.511^(c)^
	Weakness/acroanesthesia (no/yes)	112 (43.2)/147 (56.8)	81 (43.1)/107 (56.9)	9 (40.9)/13 (59.1)	22 (44.9)/27 (55.1)	0.949^(c)^
RAAS factors	PAC (lying position) (ng/dL)	30.65 (18.87)	32.94 (22.88)	29.59 (12.89)	25.53 (15.89)	**0.004**^(b)^
	PAC (standing position) (ng/dL)	32.87 (19.17)	34.73 (20.51)	31.056 (14.06)	28.27 (21.20)	0.084^(b)^
	PRA (lying position) (ng/ml/h)	0.10 (0.11)	0.10 (0.11)	0.05 (0.16)	0.10 (0.11)	0.478^(b)^
	PRA (standing position) (ng/ml/h)	0.14 (0.45)	0.12 (0.43)	0.26 (0.82)	0.26 (0.89)	0.543^(b)^
	ARR (lying position)	333.91 (540.35)	379.85 (533.55)	451.20 (699.61)	189.00 (338.66)	0.093^(b)^
	ARR (standing position)	183.79 (445.02)	213.58 (456.68)	162.53 (447.44)	120.44 (380.59)	0.179^(b)^
Serum electrolytes	Random serum Na (mmol/L)	143.62 ± 2.56	143.61 ± 2.53	144.68 ± 2.18	143.20 ± 2.73	0.077^(a)^
	Random serum K (mmol/L)	3.23 ± 0.63	3.19 ± 0.63	3.21 ± 0.72	3.40 ± 0.58	0.107^(a)^
	Random serum Cl (mmol/L)	102.59 ± 2.78	102.66 ± 2.73	103.00 ± 3.23	102.13 ± 2.74	0.383^(a)^
	Random serum Ca (mmol/L)	2.23 ± 0.22	2.24 ± 0.12	2.26 ± 0.12	2.17 ± 0.45	0.115^(a)^
	Random serum Mg (mmol/L)	0.87 ± 0.15	0.86 ± 0.09	0.90 ± 0.08	0.91 ± 0.29	0.131^(a)^
	Random serum P (mmol/L)	1.01 ± 0.20	1.00 ± 0.19	1.03 ± 0.23	1.01 ± 0.22	0.805^(a)^
	Minimal serum K (mmol/L)	2.76 ± 0.63	2.75 ± 0.63	2.74 ± 0.72	2.80 ± 0.60	0.885^(a)^
Urinary electrolytes	24 h Urinary K (mmol/24 h)	57.64 ± 27.88	56.67 ± 27.77	64.13 ± 37.29	56.89 ± 20.08	0.558^(a)^
	24 h Urinary Na (mmol/24 h)	144.12 ± 69.61	142.22 ± 72.28	129.34 ± 56.65	162.04 ± 65.61	0.258^(a)^
	24 h Urinary Cl (mmol/24 h)	140.65 ± 68.58	139.31 ± 70.73	133.14 ± 72.19	151.23 ± 57.66	0.637^(a)^
	24 h Urinary Ca (mmol/24 h)	5.79 ± 2.42	5.49 ± 2.28	6.20 ± 2.93	6.85 ± 2.39	**0.044**^(a)^
	24 h Urinary Mg (mmol/24 h)	3.42 ± 1.30	3.31 ± 1.22	3.34 ± 1.52	3.94 ± 1.39	0.114^(a)^
	24 h Urinary P (mmol/24 h)	17.87 ± 8.61	16.65 ± 6.21	17.79 ± 6.44	23.29 ± 15.20	**0.004**^(a)^
	CONC of Urinary K (mmol/L)	76.69 ± 34.61	77.23 ± 36.62	71.56 ± 29.78	77.93 ± 30.51	0.767^(a)^
	CONC of Urinary Na (mmol/L)	76.15 ± 36.33	77.68 ± 39.03	72.31 ± 31.42	73.21 ± 29.25	0.715^(a)^
	CONC of Urinary Cl (mmol/L)	3.28 ± 1.51	3.16 ± 1.43	3.17 ± 1.41	3.81 ± 1.77	0.108^(a)^
	CONC of Urinary Ca (mmol/L)	1.96 ± 0.85	1.94 ± 0.78	1.74 ± 0.93	2.21 ± 1.04	0.152^(a)^
	CONC of Urinary Mg (mmol/L)	10.32 ± 5.64	10.41 ± 6.27	9.28 ± 3.48	10.62 ± 4.08	0.697^(a)^
	CONC of Urinary P (mmol/L)	76.69 ± 34.61	77.23 ± 36.62	71.56 ± 29.78	77.93 ± 30.51	0.767^(a)^
Glucocorticoid metabolic factors	PTC8 (nmol/L)	373.70 (197.50)	367.25 (206.08)	393.00 (186.30)	377.15 (240.25)	0.875^(b)^
	PTC0 (nmol/L)	69.90 (70.91)	68.83 (65.83)	89.53 (91.86)	72.61 (97.28)	0.540^(b)^
	ACTH (ng/L)	22.74 (21.85)	22.74 (19.42)	20.11 (30.06)	20.31 (25.77)	0.720^(b)^
	24 h UFC (μg/24 h)	79.20 (56.73)	84.40 (54.35)	75.50 (43.30)	68.85 (72.20)	0.766^(b)^
	CONC of 24 h UFC (μg/L)	79.20 (56.73)	44.00 (38.93)	39.85 (22.11)	41.69 (32.56)	0.369^(b)^
Lipid metabolic factors	TG (mmol/L)	1.15 (0.94)	1.09 (0.91)	1.21 (1.40)	1.49 (1.39)	**0.003**^(b)^
	CHOL (mmol/L)	4.33 ± 0.89	4.41 ± 0.89	4.12 ± 0.76	4.12 ± 0.89	0.054^(a)^
	HDL (mmol/L)	1.27 (0.46)	1.31 (0.50)	1.23 (0.39)	1.09 (0.43)	**<0.001**^(b)^
	LDL (mmol/L)	2.51 ± 0.79	2.59 ± 0.81	2.27 ± 0.63	2.27 ± 0.72	**0.014**^(a)^
Renal function factors	Creatinine (μmol/L)	62.00 (29.00)	59.00 (27.75)	67.00 (38.50)	73.00 (25.50)	**0.008**^(b)^
	eGFR (mL/min/1.73 *m*^2^)	103.42 (23.80)	107.68 (21.95)	99.10 (26.25)	96.71 (22.80)	**0.001**^(b)^
	Uric acid (μmol/L)	313.88 ± 93.89	301.99 ± 92.78	347.14 ± 102.22	344.56 ± 85.16	**0.004**^(a)^
	BUN (mmol/L)	4.35 (1.80)	4.30 (1.80)	4.32 (1.40)	4.55 (1.80)	0.143^(b)^
Glucose metabolic factors	RBG (mmol/L)	4.90 (1.02)	4.72 (0.72)	5.00 (1.42)	6.18 (2.07)	**<0.001**^(b)^
	OGTT0 (mmol/L)	5.32 ± 1.38	4.74 ± 0.49	4.89 ± 0.45	6.90 ± 1.79	**<0.001**^(a)^
	OGTT120 (mmol/L)	9.74 ± 4.58	6.31 ± 0.81	9.19 ± 0.86	15.89 ± 3.72	**<0.001**^(a)^
	HbAC1 (%)	6.01 ± 1.22	5.24 ± 0.44	5.41 ± 0.34	6.96 ± 1.29	**<0.001**^(a)^

*PA, primary aldosteronism; DM, diabetes mellitus; IFG, impaired fasting glucose; IGT, impaired glucose tolerance; RAAS, renin-angiotensin-aldosterone system; BMI, body mass index; SBP, systolic blood pressure; PAC, plasma aldosterone concentration; PRA, plasma renin activity; ARR, aldosterone-to-renin ratio; K, potassium; Na, sodium; Cl, chlorine; Ca, calcium; Mg, magnesium; P, phosphorus; CONC, concentration; PTC8, plasma total cortisol at 8 am; PTC0, plasma total cortisol at 0 am; ACTH, adrenocorticotropin; 24 h UFC, total 24 h urine free cortisol; TG, triglycerides; CHOL, total cholesterol; HDL, high density lipoprotein; LDL, low density lipoprotein; eGFR, estimated glomerular filtration rate; BUN, blood urea nitrogen; RBG, random blood glucose; OGTT0, 0-minute oral glucose tolerance test; OGTT120, 120-minute oral glucose tolerance test; HbAC1, glycosylated hemoglobin*.

(a) *Analysis of variance (ANOVA)*.

(b) *Kruskal-Wallis rank sum test*.

(c) *Chi-square test*.

*Bold values indicate that the differences are statistically different (p < 0.05)*.

Clinical characteristics and laboratory values were also compared between three groups. PA patients with DM were older and fatter. They had also suffered from longer period of hypertension before the diagnosis of PA. Males were more likely to develop DM. When it came to renin-angiotensin-aldosterone system factors, it was interesting that no matter in which position (lying or standing), PAC and ARR were lower and plasma renin activity were higher in PA + DM group. However, only PAC of lying position was statistically different (*p* = 0.004). The concentration of serum electrolytes was not different between groups. The total 24 h urinary Ca and P were much higher in DM patients. Glucocorticoid metabolic factors did not have statistical difference between three groups. In terms of lipid metabolic factors, PA patients with DM had higher TG (*p* = 0.003), lower HDL (*p* < 0.001), and lower LDL (*p* = 0.014). We also compared several parameters associated with renal function and found that creatinine (*p* = 0.008), uric acid (*p* = 0.004), and BUN (*p* = 0.143) were much higher in PA + DM group, and eGFR (*p* = 0.001) was lower. It was not surprising that the glucose level was higher in DM patients (*p* < 0.001).

Due to some clinical and biochemical parameters which differed between PA with and without DM were related to metabolic syndrome (MetS), we also compared the prevalence of MetS between three groups. The definition of MetS was referred to that updated by International Diabetes Federation (17). There were 93 (49.5%), 16 (76.2%), and 45 (91.8%) patients with MetS in PA, PA + IFG/IGT, and PA + DM groups, respectively (*p* < 0.001). Because the diagnostic criteria of MetS contained factors of blood pressure and FPG, the logistic regression analyses of MetS between PA, PA + IFG/IGT, and PA + DM groups might be biased. Thus, we only analyzed lipid metabolic factors, the parts of MetS component in the following logistic regression analyses.

### Potential Risk Factors for Dysglycemia in PA Patients

Between PA patients with and without DM, univariate logistic regression analysis revealed that many factors were associated with DM, including age, sex, BMI, duration of hypertension, minimal diameter of tumor, three lipid metabolic factors, four renal function factors, random concentration of serum potassium, concentration of 24 h urinary Ca, and total 24 h urinary Ca/Mg/P (Table 2). Many studies had already reported that older, male, fatty, and hyperlipemic people were more likely to have DM. To eliminate the bias of these confounders, like age, sex, BMI, TG, CHOL, HDL, and LDL, multivariate logistic regression analysis was also performed. Results suggested that higher maximal SBP, higher total 24 h urinary P, higher concentration of urinary Ca and higher BUN were risk factors for DM in PA patients, while higher random serum sodium were protective one (Table 2).

**Table 2:** Univariate and multivariate logistic regression analyses for risk factors of DM/IFG/IGT in PA patients.

**Factors**		**PA patients with or without DM**				**PA patients with or without DM/IFG/IGT**			
		**Univariate**		**Multivariate**		**Univariate**		**Multivariate**	
		**OR**	***P*-value**	**OR**	***P*-value**	**OR**	***P*-value**	**OR**	***P*-value**
Clinical characteristics	Age	1.070 (1.039–1.102)	**<0.001**	—	—	1.065 (1.038–1.093)	**<0.001**	—	—
	Sex (male)	2.635 (1.385–5.014)	**0.003**	—	—	2.333 (1.337–4.073)	**0.003**	—	—
	BMI	1.332 (1.202–1.476)	**<0.001**	—	—	1.266 (1.158–1.384)	**<0.001**	—	—
	Duration of hypertension	1.007 (1.003–1.011)	**0.001**	1.003 (0.997–1.008)	0.343	1.006 (1.002–1.010)	**0.002**	1.000 (0.995–1.005)	0.964
	Maximal SBP	1.010 (0.996–1.024)	0.156	1.019 (1.001–1.037)	**0.043**	1.009 (0.997–1.022)	0.145	1.017 (1.001–1.034)	**0.038**
	Maximal tumor diameter	0.548 (0.308–0.975)	**0.041**	0.657 (0.332–1.300)	0.227	0.711 (0.454–1.113)	0.135	0.817 (0.479–1.394)	0.459
	Dizziness/headache (yes)	0.929 (0.499–1.731)	0.817	0.960 (0.440–2.096)	0.919	0.905 (0.524–1.563)	0.721	0.836 (0.418–1.673)	0.613
	Palpitation (yes)	1.125 (0.543–2.328)	0.751	0.861 (0.342–2.168)	0.751	1.352 (0.719–2.541)	0.349	1.279 (0.574–2.848)	0.547
	Weakness/acroanesthesia (yes)	0.920 (0.492–1.721)	0.795	0.742 (0.345–1.598)	0.446	0.977 (0.563–1.694)	0.933	0.776 (0.393–1.532)	0.465
RAAS factors	PAC (lying position)	0.992 (0.973–1.012)	0.436	0.996 (0.981–1.012)	0.658	0.984 (0.965–1.004)	0.108	0.993 (0.978–1.008)	0.360
	PAC (standing position)	1.004 (0.993–1.015)	0.516	1.002 (0.988–1.016)	0.803	1.000 (0.989–1.012)	0.948	0.999 (0.987–1.012)	0.892
	PRA (lying position)	0.834 (0.427–1.631)	0.596	0.890 (0.344–2.299)	0.809	0.709 (0.318–1.581)	0.400	0.669 (0.234–1.917)	0.454
	PRA (standing position)	1.044 (0.836–1.303)	0.706	0.971 (0.679–1.388)	0.871	1.090 (0.894–1.328)	0.394	1.069 (0.844–1.354)	0.579
	ARR (lying position)	0.999 (1.000–0.345)	0.091	1.000 (0.999–1.000)	0.389	1.000 (0.999–1.000)	0.345	1.000 (1.000–1.001)	0.761
	ARR (standing position)	1.000 (0.999–1.000)	0.300	1.000 (0.999–1.001)	0.743	1.000 (0.999–1.000)	0.455	1.000 (1.000–1.001)	0.797
Serum electrolytes	Random serum Na	0.923 (0.817–1.043)	0.199	0.827 (0.701–0.976)	**0.025**	1.008 (0.906–1.121)	0.888	0.944 (0.817–1.090)	0.432
	Random serum K	1.687 (1.033–2.755)	**0.037**	1.492 (0.804–2.767)	0.205	1.463 (0.948–2.258)	0.085	1.28 (0.743–2.207)	0.374
	Random serum Cl	0.930 (0.831–1.04)	0.204	0.891 (0.773–1.026)	0.109	0.968 (0.877–1.068)	0.512	0.933 (0.824–1.057)	0.279
	Random serum Ca	0.331 (0.095–1.155)	0.083	0.682 (0.163–2.849)	0.600	0.478 (0.149–1.531)	0.214	1.038 (0.273–3.943)	0.957
	Random serum Mg	4.264 (0.671–27.109)	0.124	1.760 (0.189–16.404)	0.620	6.026 (0.797–45.537)	0.082	5.073 (0.225–114.238)	0.307
	Random serum P	1.045 (0.221–4.936)	0.956	3.251 (0.464–22.805)	0.235	1.369 (0.352–5.323)	0.650	3.430 (0.610–19.277)	0.162
	Minimal serum K	1.133 (0.691–1.857)	0.620	1.234 (0.678–2.245)	0.492	1.085 (0.702–1.676)	0.714	1.324 (0.775–2.262)	0.305
Urinary electrolytes	24 h Urinary K	0.999 (0.984–1.014)	0.877	0.998 (0.979–1.017)	0.823	1.004 (0.992–1.016)	0.514	1.009 (0.993–1.025)	0.254
	24 h Urinary Na	1.004 (0.999–1.010)	0.144	1.002 (0.994–1.010)	0.608	1.001 (0.996–1.006)	0.606	0.999 (0.992–1.006)	0.828
	24 h Urinary Cl	1.003 (0.997–1.009)	0.377	1.000 (0.992–1.008)	0.975	1.001 (0.996–1.006)	0.713	0.999 (0.993–1.006)	0.867
	24 h Urinary Ca	1.230 (1.02–1.483)	**0.030**	1.172 (0.953–1.442)	0.133	1.204 (1.028–1.409)	**0.021**	1.230 (1.005–1.504)	**0.044**
	24 h Urinary Mg	1.410 (1.012–1.965)	**0.043**	1.351 (0.887–2.057)	0.161	1.239 (0.934–1.643)	0.137	1.245 (0.861–1.801)	0.244
	24 h Urinary P	1.086 (1.017–1.160)	**0.014**	1.079 (1.000–1.165)	**0.050**	1.065 (1.008–1.126)	**0.024**	1.080 (1.000–1.166)	**0.050**
	CONC of Urinary K	0.973 (0.945–1.002)	0.067	0.969 (0.932–1.007)	0.109	0.984 (0.962–1.005)	0.138	0.984 (0.957–1.012)	0.264
	CONC of Urinary Na	1.001 (0.991–1.012)	0.813	1.000 (0.987–1.014)	0.996	0.999 (0.989–1.008)	0.762	0.999 (0.987–1.010)	0.804
	CONC of Urinary Cl	0.997 (0.987–1.008)	0.592	0.995 (0.982–1.009)	0.471	0.996 (0.987–1.005)	0.414	0.995 (0.984–1.006)	0.396
	CONC of Urinary Ca	1.309 (1.014–1.689)	**0.039**	1.449 (1.043–2.013)	**0.027**	1.193 (0.953–1.492)	0.124	1.266 (0.953–1.681)	0.103
	CONC of Urinary Mg	1.462 (0.939–2.275)	0.093	1.490 (0.826–2.688)	0.186	1.132 (0.763–1.679)	0.538	1.057 (0.641–1.744)	0.828
	CONC of Urinary P	1.011 (0.944–1.011)	0.748	1.037 (0.950–1.133)	0.411	0.990 (0.930–1.054)	0.756	1.002 (0.925–1.084)	0.969
Glucocorticoid metabolic factors	PTC8	1.000 (0.998–1.002)	0.758	1.001 (0.998–1.003)	0.672	0.999 (0.998–1.001)	0.406	1.000 (0.998–1.002)	0.999
	PTC0	1.003 (0.998–1.008)	0.221	1.002 (0.996–1.008)	0.547	1.003 (0.998–1.007)	0.243	1.001 (0.995–1.007)	0.696
	ACTH	0.998 (0.978–1.018)	0.807	0.990 (0.965–1.015)	0.424	1.000 (0.983–1.017)	0.969	1.000 (0.978–1.023)	0.999
	24 h UFC	0.999 (0.993–1.006)	0.870	1.002 (0.993–1.010)	0.726	0.998 (0.992–1.004)	0.428	1.009 (0.992–1.007)	0.883
	CONC of 24 h UFC	0.989 (0.975–1.004)	0.141	1.981 (0.620–6.325)	0.248	0.987 (0.974–1.000)	**0.042**	1.990 (0.974–1.005)	0.189
Lipid metabolic factors	TG	1.308 (1.043–1.641)	**0.020**	—	—	1.266 (1.017–1.576)	**0.035**	—	—
	CHOL	0.699 (0.483–1.011)	0.057	—	—	0.672 (0.485–0.931)	**0.017**	—	—
	HDL	0.157 (0.055–0.445)	**<0.001**	—	—	0.240 (0.102–0.564)	**0.001**	—	—
	LDL	0.612 (0.401–0.935)	**0.023**	—	—	0.576 (0.396–0.839)	**0.004**	—	—
Renal function factors	Creatinine	1.018 (1.004–1.031)	**0.012**	1.006 (0.985–1.027)	0.586	1.017 (1.005–1.030)	**0.007**	1.005 (0.986–1.024)	0.629
	eGFR	0.974 (0.959–0.990)	**0.001**	1.000 (0.974–1.026)	0.994	0.976 (0.962–0.990)	**0.001**	1.004 (0.980–1.027)	0.767
	Uric acid	1.004 (1.001–1.008)	**0.012**	1.000 (0.994–1.005)	0.891	1.005 (1.002–1.008)	**0.001**	1.003 (0.998–1.008)	0.230
	BUN	1.247 (1.052–1.478)	**0.011**	1.243 (1.007–1.533)	**0.043**	1.143 (0.978–1.335)	0.093	1.066 (0.880–1.292)	0.512

*PA, primary aldosteronism; DM, diabetes mellitus; IFG, impaired fasting glucose; IGT, impaired glucose tolerance; RAAS, renin-angiotensin-aldosterone system; BMI, body mass index; SBP, systolic blood pressure; PAC, plasma aldosterone concentration; PRA, plasma renin activity; ARR, aldosterone-to-renin ratio; K, potassium; Na, sodium; Cl, chlorine; Ca, calcium; Mg, magnesium; P, phosphorus; CONC, concentration; PTC8, plasma total cortisol at 8 am; PTC0, plasma total cortisol at 0 am; ACTH, adrenocorticotropin; 24 h UFC, total 24 h urine free cortisol; TG, triglycerides; CHOL, total cholesterol; HDL, high density lipoprotein; LDL, low density lipoprotein; eGFR, estimated glomerular filtration rate; BUN, Bold values indicate that the differences are statistically different (p < 0.05)*.

Similar factors were shown to be related to DM/IFG/IGT after univariate logistic regression analyses. We also performed multivariate logistic regression analysis and found that PA patients with higher maximal SBP, higher total 24 h urinary Ca and higher total 24 h urinary P were more prone to DM/IFG/IGT (Table 2).

### Correlation Between RBG and Potential Risk Factors for Dysglycemia in PA Patients

**Figure 1:**
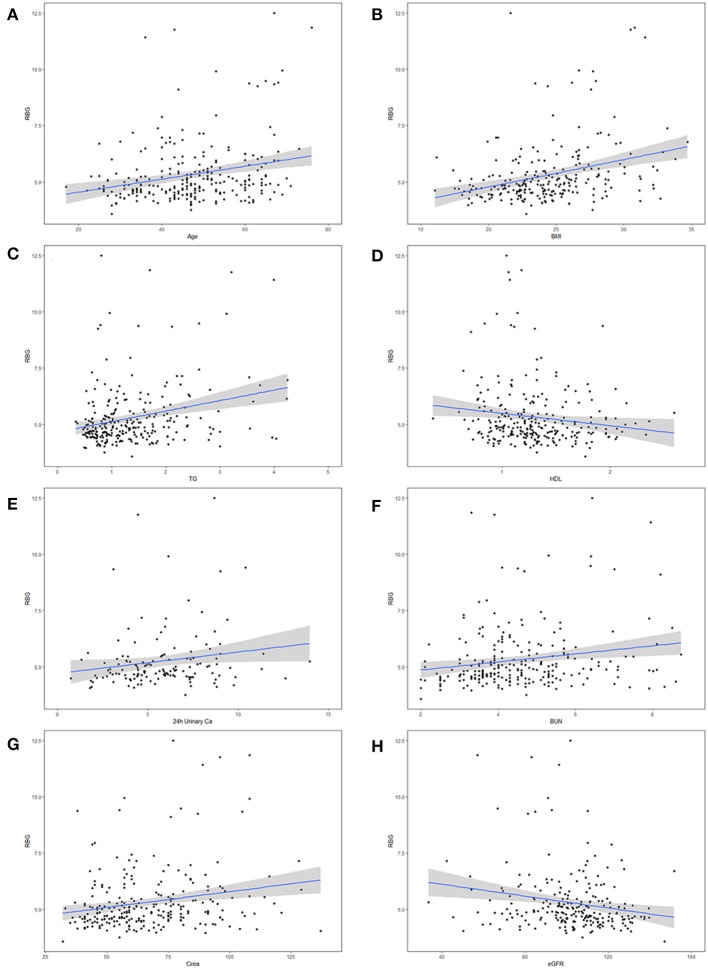
Correlation between maximal random blood glucose (RBG) and potential risk factors for diabetes mellitus (DM) in primary aldosteronism (PA) patients. The risk factors including **(A)** age, **(B)** body mass index (BMI), **(C)** triglycerides (TG), **(D)** high density lipoprotein (HDL), **(E)** 24 h urinary Calcium, **(F)** blood urea nitrogen (BUN), **(G)** serum creatinine (Crea), and **(H)** estimated glomerular filtration rate (eGFR).

Pearson correlation analysis suggested that age, BMI, maximal SBP, total 24 h urinary Ca, total 24 h urinary Mg, concentration of urinary Ca, concentration of urinary Mg, TG, CHOL, creatinine, uric acid and BUN were positively correlated with RBG. On the contrary, there was a negative correlation between RBG and random serum chlorine, HDL as well as eGFR (Table 3). Figure 1 give a visual representation of some relationships.

**Table 3:** Pearson correlation between potential risk factors of dysglycemia and RBG.

	**Factors**	***r***	***P*-value**
Clinical characteristics	Age (years)	0.255	**<0.001**
	BMI (kg/m^2^)	0.317	**<0.001**
	Duration of hypertension (months)	0.110	0.082
	Maximal SBP (mm Hg)	0.152	**0.016**
	Maximal diameter of tumor (cm)	−0.122	0.060
	Dizziness or headache (no/yes)	−0.004	0.945
	Palpitation (no/yes)	0.093	0.137
	Weakness or acroanesthesia (no/yes)	0.014	0.824
RAAS factors	PAC (lying position) (ng/dL)	−.126	0.057
	PAC (standing position) (ng/dL)	−0.055	0.393
	PRA (lying position) (ng/ml/h)	−0.062	0.335
	PRA (standing position) (ng/ml/h)	−0.030	0.644
	ARR (lying position)	−0.081	0.229
	ARR (standing position)	−0.002	0.970
Serum electrolytes	Random serum Na (mmol/L)	−0.086	0.169
	Random serum K (mmol/L)	0.008	0.898
	Random serum Cl (mmol/L)	−0.150	**0.016**
	Random serum Ca (mmol/L)	0.014	0.824
	Random serum Mg (mmol/L)	−0.044	0.488
	Random serum P (mmol/L)	−0.090	0.149
	Minimal serum potassium (mmol/L)	0.045	0.483
Urinary electrolytes	24 h Urinary K (mmol/24 h)	−0.019	0.819
	24 h Urinary Na (mmol/24 h)	0.090	0.268
	24 h Urinary Cl (mmol/24 h)	0.061	0.451
	24 h Urinary Ca (mmol/24 h)	0.172	**0.044**
	24h Urinary Mg (mmol/24 h)	0.200	**0.019**
	24 h Urinary P (mmol/24 h)	0.069	0.428
	CONC of Urinary K (mmol/L)	−0.088	0.247
	CONC of Urinary Na (mmol/L)	−0.021	0.777
	CONC of Urinary Cl (mmol/L)	−0.038	0.612
	CONC of Urinary Ca (mmol/L)	0.165	**0.039**
	CONC of Urinary Mg (mmol/L)	0.165	**0.039**
	CONC of Urinary P (mmol/L)	0.080	0.325
Glucocorticoid metabolic factors	PTC−8 (Cortisol) (nmol/L)	0.024	0.726
	PTC−0 (Cortisol) (nmol/L)	0.018	0.807
	ACTH (ng/L)	0.059	0.410
	Total 24 h UFC (μg/24 h)	0.059	0.430
	CONC of 24 h UFC (μg/L)	−0.057	0.454
Lipid metabolic factors	TG (mmol/L)	0.238	**<0.001**
	CHOL (mmol/L)	0.258	**<0.001**
	HDL (mmol/L)	−0.135	**0.029**
	LDL (mmol/L)	0.012	0.854
Renal function factors	Creatinine (μmol/L)	0.194	**0.002**
	eGFR (mL/min/1.73 *m*^2^)	−0.192	**0.003**
	Uric acid (μmol/L)	0.216	**<0.001**
	BUN (mmol/L)	0.137	**0.028**

*RBG, random blood glucose; RAAS, renin-angiotensin-aldosterone system; BMI, body mass index; SBP, systolic blood pressure; PAC, plasma aldosterone concentration; PRA, plasma renin activity; ARR, aldosterone-to-renin ratio; K, potassium; Na, sodium; Cl, chlorine; Ca, calcium; Mg, magnesium; P, phosphorus; CONC, concentration; PTC8, plasma total cortisol at 8 am; PTC0, plasma total cortisol at 0 am; ACTH, adrenocorticotropin; 24 h UFC, total 24 h urine free cortisol; TG, triglycerides; CHOL, total cholesterol; HDL, high density lipoprotein; LDL, low density lipoprotein; eGFR, estimated glomerular filtration rate; BUN, blood urea nitrogen. Bold values indicate that the differences are statistically different (p < 0.05)*.

### Correlation Between PAC, BUN, and Potential Associated Factors

A Pearson correlation analysis was conducted between PAC (lying position) and potential biases which affected the difference of PAC between three groups, such as age and BMI. There was a negative correlation between PAC (lying position) and age (*p* = 0.003). In addition, Pearson correlation analysis revealed that the level of BUN was positively correlated with creatinine (*r* = 0.622, *p* < 0.001) and negatively with eGFR (*r* = −0.572, *p* < 0.001) (Supplementary Figure 1).

### The Verification of Highly Potential Risk Factors for Dysglycemia in PA Patients

Based on the above-mentioned results, PA patients with the following parameters were more likely to develop DM: older age, male, higher BMI, higher TG, lower HDL, higher BUN and higher total 24 h urinary Ca. We divided PA patients into two subsamples by cutoff values of these parameters, and then compared the percentage of DM or DM/IFG/IGT between them. According to the BUN (< upper limit of normal or ≥ upper limit of normal), we separated PA patients into two parts. The upper limit of normal of BUN for male and female were 8.22 and 7.70 mmol/L, respectively. Chi-square test revealed that prevalence of DM in the 259 PA patients were significantly different between two parts (17.4 vs. 45.5%, *p* = 0.035). However, there was no difference in the prevalence of DM/IFG/IGT between these two parts (*p* = 0.175). In addition, patients were also divided into two subsamples by 24 h urinary Ca level. We found that DM patients (32.3 vs. 11.3%, *p* = 0.010) or DM/IFG/IGT patients (45.2 vs. 23.6%, *p* = 0.025) were more abundant in group with 24 h urinary Ca > 7.5 mmol/24 h (Table 4).

**Table 4:** The effect of age, sex, BMI, TG, HDL, BUN, and 24 h urinary Ca on the glucose metabolism.

	**PA patients with or without DM**			**PA patients with or without DM/IFG/IGT**		
	**Without DM**	**With DM**	***P*-value**	**Without DM/IFG/IGT**	**With DM/IFG/IGT**	***P*-value**
Age <50	141 (89.2%)	17 (10.8%)	**<0.001**	131 (82.9%)	27 (17.1%)	**<0.001**
Age ≥ 50	69 (68.3%)	32 (31.7%)		57 (56.4%)	44 (43.6%)	
Female	127 (87.6%)	18 (12.4%)	**0.004**	116 (80.0%)	29 (20.0%)	**0.003**
Male	83 (72.8%)	31 (27.2%)		72 (63.2%)	42 (36.8%)	
BMI <24	109 (93.2%)	8 (6.8%)	**<0.001**	103 (88.0%)	14 (12.0%)	**<0.001**
BMI ≥ 24	85 (67.5%)	41 (32.5%)		72 (57.1%)	54 (42.9%)	
TG ≤ 1.83 (mmol/L)	167 (73.1%)	34 (16.9%)	0.132	152 (75.6%)	49 (24.4%)	**0.046**
TG > 1.83 (mmol/L)	43 (74.1%)	15 (25.9%)		36 (62.1%)	22 (37.9%)	
HDL ≤ 0.9 (mmol/L)	20 (60.6%)	13 (39.4%)	**0.003**	17 (51.5%)	16 (48.5%)	**0.006**
HDL>0.9 (mmol/L)	190 (84.1%)	36 (15.9%)		171 (75.7%)	55 (24.3%)	
BUN < ULN	204 (82.6%)	43 (17.4%)	**0.035**	182 (73.7%)	65 (26.3%)	0.175
BUN ≥ ULN	6 (54.5%)	5 (45.5%)		6 (54.5%)	5 (45.5%)	
24 h Urinary Ca ≤ 7.5 (mmol/24 h)	94 (88.7%)	12 (11.3%)	**0.010**	81 (76.4%)	25 (23.6%)	**0.025**
24 h Urinary Ca > 7.5 (mmol/24 h)	21 (67.7%)	10 (32.3%)		17 (54.8%)	14 (45.2%)	

*PA, primary aldosteronism; DM, diabetes mellitus; IFG, impaired fasting glucose; IGT, impaired glucose tolerance; BMI, body mass index; TG, triglycerides; HDL, high density lipoprotein; BUN, blood urea nitrogen; Ca, calcium; ULN, upper limit of normal. Bold values indicate that the differences are statistically different (p < 0.05)*.

### Nomogram Model for Predicting DM in PA Patients

**Figure 2:**
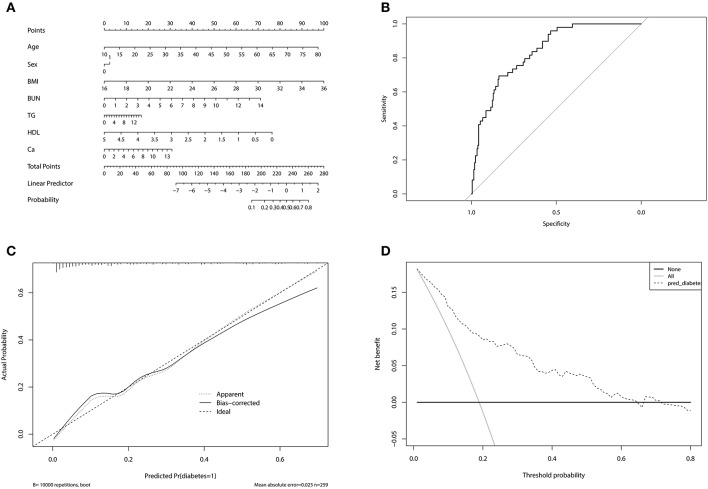
**(A)** Nomogram for predicting diabetes mellitus in primary aldosteronism patients. In sex, “0” represented female, “1” represented male. **(B)** The receiver operating characteristic of the model. The upper left point of the curve had the highest sensitivity and specificity at the same time, and the corresponding risk was the suitable cutoff value for predicting the probability of diabetes mellitus in primary aldosteronism patients. **(C)** Calibration curve of the nomogram. The dashed line was the ideal prediction and the solid line was the bias-corrected predictive performance of the nomogram. The closer the solid line fitted to the dashed line, the better the predictive accuracy of the nomogram was. **(D)** Decision curve analysis for the radiomics nomogram. The dashed line represented the nomogram. The gray line was the hypothesis that all primary aldosteronism patients had diabetes mellitus. The black line was the hypothesis that no primary aldosteronism patients had diabetes mellitus.

A novel nomogram model was developed to predict the probability of DM in PA patients (Figure 2A). The factors used in the model included sex, age, BMI, BUN, TG, HDL, and 24 h urinary Ca. The model had a good predictive accuracy, with a ROC AUC of 0.839 (95% CI 0.784–0.893) (Figure 2B). When the risk of DM was higher than the cutoff value of 0.209, the sensitivity and specificity were 0.735 and 0.757, respectively. The internal 10-fold cross validation was also performed, obtaining a mean ROC AUC of 0.809 (95% CI: 0.087–0.812). Calibration curve of the nomogram indicated that the predictive accuracy of the nomogram was good (Figure 2C) population. The decision curve analysis showed that when the probability of DM is between 0.10 and 0.65, and clinicians just treat DM of PA patients with probability of DM > 0.209, the net benefit was higher than “treat-all” and “treat-none” options (Figure 2D).

## Discussion

The study showed that the prevalence rate of DM was 18.9% in PA patients in West China Hospital and it was higher than that in general Chinese population (5.5–9.1%) (18–20). Additionally, nearly three quarters of DM patients had hypertension previously. Similarly, Hanslik et al. reported that DM was more common in PA patients than in the control population matched with sex, age, BMI and blood pressure (17.2 vs. 10.4%) (8). 16.0% of PA patients were also diagnosed with DM, and only 10.9% of patients with EH had diabetes (13). When compared with general population (12.1%) and EH patients (14.3%), higher percentage of DM was also seen in PA patients (21.6%) in Japan (9). Based on these data, it was obvious that PA patients are more likely to develop DM.

The etiology of high prevalence rate of DM in PA patients was still unclear. In our study, we did not find any association between RBG and PAC, plasma renin activity or ARR regardless of position when blood sample was collected. Multivariate logistic analysis also did not reveal any difference in these renin-angiotensin-aldosterone system factors between PA patients with various level of serum glucose. Some scientists reported that PAC was higher in PA patients with DM than those without DM (10–12). They proposed that excessive aldosterone in blood may impair the structure and function of the pancreatic beta-cells by inducing inflammatory and oxidative stress reaction, followed by decreased insulin release. In addition, it was also shown *in vitro* that aldosterone may inhibit extra-renal tissues (such as adipocytes and skeletal muscle cells) from absorbing glucose by degrading insulin receptors via increasing proinflammatory cytokines and reactive oxygen species (21, 22). However, some recent studies did not find any evidence to support that higher PAC may have adverse effect on glucose metabolism (9, 13, 14). Interestingly, our study found that PA + DM patients had lower PAC (lying position) when compared with PA patients. The most likely reason may be the existence of biases. We found a negative correlation between PAC (lying position) and age. In addition, multivariate logistic regression analysis showed that PAC (lying position) did not influence the probability of having DM. Taken together, these results indicated that DM was more likely to appear in the elderly, instead of patients with lower PAC (lying position).

Some studies reported that hypokalemia may be the risk factor for impaired glucose metabolism because of lower serum potassium concentration in PA patients with DM than in those without DM (11, 12). Researchers thought that hypokalemia may decrease insulin secretion and induce insulin resistance, followed by dysbiosis of glucose metabolism. However, our results did not support these opinions because there was no correlation between the level of serum potassium and RBG, which was also seen in other studies (9, 10, 13).

In our study, we found that older, fatter PA patients and those with dyslipidemia or higher blood pressure may have higher likelihood of getting DM, which had already been elucidated by some epidemiologic studies of DM. In addition, the percentage of MetS was much higher in PA + DM group when compared with PA group. These evidences indicated that MetS, especially dyslipidemia was an important risk factor for DM. One accepted reason is that cytokine release/JNK signaling from adipose tissue caused low-grade inflammation associated with inflammasomes (23). Then lipid-induced insulin resistance in skeletal muscle occurs, which stems from deficiency in insulin-stimulated glucose transport activity. Because of inhibition of hepatic glucose production and stimulation of glycogen synthesis, resistance to insulin in steatotic livers also occurred (24). In addition, recent studies found that fat deposition and amylin deposition in the pancreas may result in early pancreatic beta-cell dysfunction, followed by insulin resistance and MetS (23, 25). On the other hand, PA may also contribute to the development of MetS, especially dyslipidemia. Some studies reported that PA patients had a higher prevalence of MetS compared with EH (40–45 vs. 30%) (26, 27). There was a positive correlation between aldosterone and TG as well as LDL levels, and a negative correlation between aldosterone and HDL (28). Thus, MetS and dyslipidemia may also be the results of PA, which coexisted with DM or then contributed to the development of DM.

It was also reported that PA could affect glucocorticoid system (29). Higher level of cortisone could be conducive to DM (23). However, our study did not find any difference of glucocorticoid metabolic factors between PA, PA + IFG/IGT, and PA + DM patients. Thus, the effect of glucocorticoid perturbation on DM was still unclear.

BUN was positively correlated with RBG. Multivariate logistic regression analysis also showed that elevated BUN was a risk factor for DM in PA patients. In addition, more PA patients with BUN ≥ upper limit of normal (45.5%) were diagnosed with DM than those with BUN < upper limit of normal (17.4%). Thus, we hypothesize that higher BUN accompanied with PA may contribute to the dysbiosis of glucose. Some experimental studies found that higher levels of BUN in mice may lead to increased reactive oxygen species, followed by decreased insulin sensitivity in adipocytes. Besides, in mice of chronic kidney disease, impaired glucose-stimulated insulin secretion was observed (30). Insulin secretion was also down-regulated in pancreatic islets cultured with urea, the concentration of which was similar to that of patients with chronic kidney disease (31). Additionally, Xie et al. also reported the association of higher BUN with increased risk of incident DM (32). However, no study has studied why BUN increased in PA patients. We think the most possible reason was impaired renal function. To verify this, we performed Pearson correlation analysis and found that the level of BUN was positively correlated with creatinine and negatively with eGFR. Although most patients' creatinine and eGFR were still within normal range, some studies found that the impaired renal function was masked by glomerular hyperfiltration, which was caused by chronic hypertension and the direct effect of excessive aldosterone (33, 34). The renal damages, including interstitial fibrosis, tubular atrophy, and hyaline sclerosis of the arterioles were verified by biopsy after adrenalectomy (35).

Although BUN was higher in PA patients with DM, an important point that should not be ignored was that most BUN was within the normal range. Thus, the increased BUN may not directly affect the glucose metabolism. Instead, it may be associated with other factors which may have a critical role in the formation of DM. Our results revealed that PA patients with DM would excrete more Ca from urine and the total 24 h urinary Ca was positively correlated with RBG. The higher excretion of Ca from urine in PA patients with DM may result from damaged kidney. To maintain the homeostasis of Ca, more parathyroid hormone (PTH) was secreted. Thus, the serum Ca was not statistically different between three groups and no association was observed between it and RBG. Because PA patients did not receive PTH examination routinely, we could not get this data and analyze the relationship between PTH and DM. However, some studies did find that increased PTH was associated with decreased insulin sensitivity and pancreatic beta-cell function (36, 37).

We found that the nomogram model including age, sex, BMI, TG, HDL, BUN, and 24 h urinary Ca could accurately predict the existence of DM. When patients are diagnosed as PA, clinicians can calculate the risk of having DM in the future based on the model. If the risk is higher than 0.209, the possibility of DM may be at least 70%, and the clinicians can give some suggestions to patients on preventing DM and monitor blood glucose regularly. By doing so, patients and clinicians can take actions in advance to reduce the possibility of DM in PA patients. The cost of monitoring blood glucose in PA patients with high risk of DM can also be much lower than that of treatment for DM and its complications.

Our study also had some limitations. First, it was a retrospective study. We could not obtain data before the diagnosis of DM. Thus, the conclusions regarding causality between the risk factors and dysglycemia can not be drawn. Second, we did not compare the prevalence of DM between PA patients and EH patients in our single institute. Third, DM patients were not followed up. Our next work is to find out which parameters are statistically different between DM patients with improved glucose metabolism and those without. It is very important because these factors may be useful for predicting the development of DM in PA patients. In addition, it was only hypothesized that PTH may be higher in DM patients, however, we did not get any data about PTH. Finally, all the data we analyzed were obtained when PA was diagnosed. Thus, the nomogram model for predicting DM in PA patients may not be accurate.

In conclusion, PA patients were more likely to have DM compared with general population. Apart from older age, overweight and dyslipidemia, higher BUN and excessive excretion of urinary Ca may be the new potential risk factors for DM in PA patients.

## Data Availability Statement

The datasets generated for this study are available on request to the corresponding author.

## Ethics Statement

The studies involving human participants were reviewed and approved by West China Hospital of Sichuan University Biomedical Research Ethics Committee, No. 37 Guo Xue Xiang, Chengdu, Sichuan, 610041, P.R China (IRB approval number 2019568).

## Author Contributions

YL, LZ, and ZL conceived and designed the study, collected and analyzed the data and wrote the manuscript. YM and LL analyzed the data. YZ, KW, and HL reviewed and edited the manuscript. All authors read and approved the manuscript.

### Conflict of Interest

The authors declare that the research was conducted in the absence of any commercial or financial relationships that could be construed as a potential conflict of interest.

## Supplementary Material

Supplementary Figure 1 **(A)** Correlation between plasma aldosterone concentration (PAC) and age, **(B)** Correlation between blood urea nitrogen (BUN) and creatinine (Crea), **(C)** Correlation between blood urea nitrogen (BUN) and estimated glomerular filtration rate (eGFR).

## Abbreviations

DM: diabetes mellitus
PA: primary aldosteronism
PAC: plasma aldosterone concentration
IFG: impaired fasting glucose
IGT: impaired glucose tolerance
ARR: aldosterone-to-renin ratio
RBG: random blood glucose
FPG: fasting plasma glucose
OGTT120: 120-minute oral glucose tolerance test
OGTT0: 0-minute oral glucose tolerance test
BMI: body mass index
SBP: systolic blood pressure
TG: triglycerides
CHOL: total cholesterol
HDL: high density lipoprotein
LDL: low density lipoprotein
EH: essential hypertension
eGFR: estimated glomerular filtration rate
BUN: blood urea nitrogen
Ca: calcium
Mg: magnesium
P: phosphorus
CI: confidence intervals
ROC: receiver operating characteristic
AUC: area under the curve
PTH: parathyroid hormone
MetS: metabolic syndrome.
