# Supplementary figures and images for: Higher Blood Urea Nitrogen and Urinary Calcium: New Risk Factors for Diabetes Mellitus in Primary Aldosteronism Patients
Source: Front Endocrinol (Lausanne). 2020 Feb 4;11:23. doi: 10.3389/fendo.2020.00023 (PMC7011190; doi:10.3389/fendo.2020.00023)

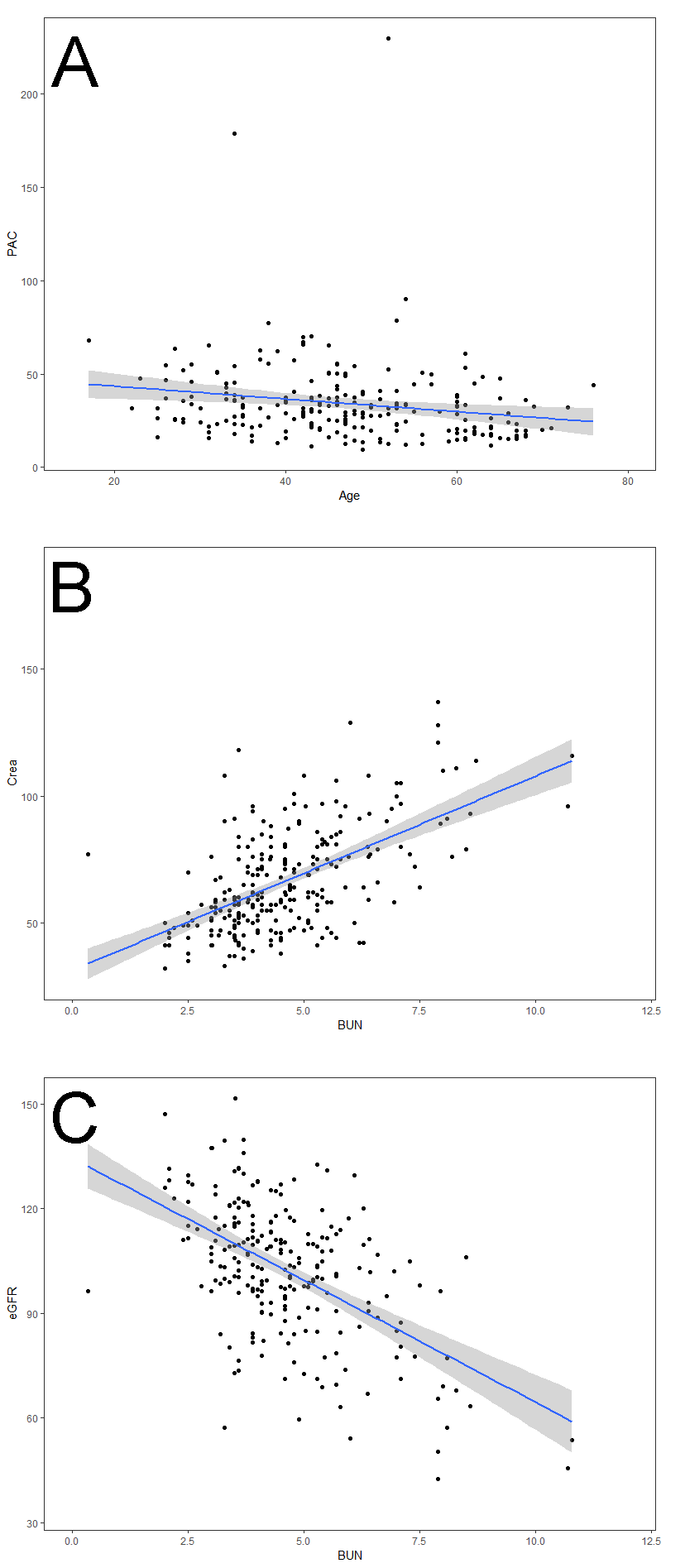

Supplement: Supplementary Figure 1 — (A) Correlation between plasma aldosterone concentration (PAC) and age, (B) Correlation between blood urea nitrogen (BUN) and creatinine (Crea), (C) Correlation between blood urea nitrogen (BUN) and estimated glomerular filtration rate (eGFR). [file Image_1.tif]
